# An unsupervised machine learning method for assessing quality of tandem mass spectra

**DOI:** 10.1186/1477-5956-10-S1-S12

**Published:** 2012-06-21

**Authors:** Wenjun Lin, Jianxin Wang, Wen-Jun Zhang, Fang-Xiang Wu

**Affiliations:** 1 Division of Biomedical Engineering, University of Saskatchewan, 57 Campus Dr., Saskatoon, S7N 5A9, Canada; 2 School of Information Science and Engineering, Central South University, Changsha, P.R.China; 3 Department of Mechanical Engineering, University of Saskatchewan, 57 Campus Dr., Saskatoon, S7N 5A9, Canada

## Abstract

**Background:**

In a single proteomic project, tandem mass spectrometers can produce hundreds of millions of tandem mass spectra. However, majority of tandem mass spectra are of poor quality, it wastes time to search them for peptides. Therefore, the quality assessment (before database search) is very useful in the pipeline of protein identification via tandem mass spectra, especially on the reduction of searching time and the decrease of false identifications. Most existing methods for quality assessment are supervised machine learning methods based on a number of features which describe the quality of tandem mass spectra. These methods need the training datasets with knowing the quality of all spectra, which are usually unavailable for the new datasets.

**Results:**

This study proposes an unsupervised machine learning method for quality assessment of tandem mass spectra without any training dataset. This proposed method estimates the conditional probabilities of spectra being high quality from the quality assessments based on individual features. The probabilities are estimated through a constraint optimization problem. An efficient algorithm is developed to solve the constraint optimization problem and is proved to be convergent. Experimental results on two datasets illustrate that if we search only tandem spectra with the high quality determined by the proposed method, we can save about 56 % and 62% of database searching time while losing only a small amount of high-quality spectra.

**Conclusions:**

Results indicate that the proposed method has a good performance for the quality assessment of tandem mass spectra and the way we estimate the conditional probabilities is effective.

## Background

Proteomics is the systematic study of proteins in order to understand their structures and functional relations [1]. One area in proteomics is to identify proteins in biological complexes via peptides identified from tandem mass spectra. Commonly used methods for identifying peptides from tandem mass spectra can be divided into two categories: database searching methods such as Mascot [2] and SEQUEST [3] and de novo sequencing methods such as PEAKS [4] and PepNovo [5]. Unfortunately, a large number of poor quality spectra are commonly observed in tandem mass spectral datasets, which contain too little, irrelevant, or ambiguous information. The existence of spectra with poor quality not only slows down the identification process, but also increases the false positives and false negatives [6]. In Keller et al's experiments [7], the mixture of 29 proteins produced 37,071 tandem mass spectra, of which only 2,784 spectra originated from those 29 proteins [8], while the rest spectra could be removed from the analysis without losing any relevant protein information. Hence, it is worthwhile to develop an automatic quality assessment algorithm to discriminate high-quality from poor-quality spectra before further interpretation.

Spectral quality assessment methods select high quality spectra for further processing, but do not change the selected spectra themselves [9]. Several spectral quality assessment methods have been developed in recent years. Existing spectral quality assessment methods generally define a number of features to describe the quality of spectra [10-15]. Based on defined features these methods assessed the quality of tandem mass spectra by supervised machine learning methods, which require labelled training datasets to train a classifier. The trained classifier is then used to classify spectra into high-quality or poor-quality ones. Ideally, the training set should be validated by some peptide identification algorithms or manual checking, i.e., the set should be correctly labelled without or with very few falsely labelled spectra. However, this information is hard to be obtained prior to the peptide identification for new dataset. Even worse, tandem mass spectrometers may produce different spectra for the same peptide under different experimental conditions. Classifier trained by one dataset may not be effective on another. Therefore, unsupervised machine learning methods are appealing for assessing the quality of tandem mass spectra. In [16], we applied the weighted k-means to classify tandem mass spectra into high-quality cluster and poor quality spectra, based on the features defined in [6].

In the literature, hundreds of features have been defined to describe the quality of tandem mass spectra, some of which are closely relevant, yet other are not. In the previous work, Ding et al [17] used a two-stage recursive feature elimination method which is based on support vector machine (SVM-RFE) to select most relevant features from those collected in the existing literature to assess the quality of tandem mass spectra. To verify the relevance of selected features, classifiers are trained with different sets of selected features and their performances are analyzed. The results demonstrate that the sets with a small number of features outperforms the full set of features, which indicates that these features together can better describe the quality of tandem mass spectra and hence improve the performance of tandem mass spectral quality assessment.

In this paper, we propose an unsupervised machine learning method with a set of 10 most relevant features from the previous work [17] to assess the quality of tandem spectra. These 10 features have clear physical meanings: the higher the individual feature value of a spectrum, the more possible it is of high quality. Therefore, each individual feature can be used to easily assign a spectrum to be of high quality or poor quality by a user specified threshold. However, the precision of assessments from each individual feature is too low. Our proposed method in this paper will integrate all assessments from 10 individual features into a consensus assessment with a better precision, based a constraint optimization model. The remainder of the paper is organized as follows. The "Method" section introduces the 10 features, describes the constraint optimization model and then present an iterative algorithm to solve it. The "Experimental results and discussion" section investigates the performance of proposed quality assessment method with two tandem mass spectra datasets with low resolution. The results are presented and discussed. The "Conclusions and future work" section concludes this study and points out some direction of the future work along with this study.

## Method

In this section, 10 features used for quality assessment of tandem mass spectra are introduced in the subsection A. In subsection B, we describe a graph-based consensus optimization method [18] to integrate individual assessments into a consensus assessment and also propose an algorithm method to solve this optimization problem. The convergence of the algorithm is also proved.

### A. Spectral features

A tandem mass spectrum usually contains tens to hundreds of m/z values with their corresponding signal intensities. In the literature, hundreds of features have been proposed to describe the quality of tandem mass spectra, for example [19-21]. In the previous study, after removing the noisy peaks by using the morphological reconstruction method [22,23], 10 most relevant spectral features are selected based on support vector machine methods [14,17] which are introduced as follows:

Feature 1 is proposed by Bern et al [15] and defined as the total normalized intensity of pairs of peaks with their m/z values summing to the mass of the precursor ion [15]. This feature is based on the reasonable assumption that the peaks with lower intensity are noises and that the complementary peaks are more likely to be signal.

Feature 2 is proposed by Flikka et al [20] and defined as the mass of uncharged precursor ion. This feature is based on the observation that most of poor quality spectra have the small mass of precursor ions as they maybe came from not long enough peptides or noisy chemical molecules.

Feature 3 is proposed by Wu et al [6] and defined as the number of peaks whose mass difference equals to one of the 20 amino acids mass (all peaks are considered as single charged). The comparison uses a tolerance which is set to 0.5 Da. This feature reflects that in the theoretical tandem mass spectrum of a peptide each of all the same type ions (for example, b-ion) in order differs an amino acid from its before- and/or after- neighbors.

Feature 4 is proposed by Flikka et al [20] and defined as the average delta mass - average of all mass differences between any two neighbor peaks in a spectrum. This feature reflects that the too-dense spectra are of poor quality [15,20,24].

Feature 5 is proposed by Bern et al [15] and called the Good-Diff Fraction which is defined as

(1) $$\text{GoodDiffs}=\sum \left\{\text{Norm}I\left(x\right)+\text{Norm}I\left(y\right)|M\left(x\right)-M\left(y\right)\approx M_{i}\text{ for some }i=1,2,\;\;\ldots ,20\right\}$$

where *M *(*x*) is the m/z value of peak *x *and *M*_1_,*M*_2_,...,*M*_20 _represents the masses of 20 amino acids (not all of which are unique). The comparison implied by ≈ uses a tolerance, which was set to 0.5 Da. Similar to Feature 3, it measures how likely two peaks are to differ by the mass of an amino acid.

Feature 6 is proposed by Wu et al [6] and defined as the number of pairs of complementary peaks. A pair of peaks is complementary if the sum of their m/z values is equal to the mass of the precursor ion (all peaks are considered as single charged). This feature measures how likely an N-terminus ion and a C-terminus ion in the tandem mass spectra are produced as the peptide fragments from the same peptide bond.

Feature 7 is proposed by Wu et al [6] and defined as the number of pairs of peaks whose m/z value differences is equal to the mass of a water molecule or an ammonia molecule (all peaks are considered as single charged). This feature measures how likely one ion in a peptide tandem mass spectrum is produced by losing a water or ammonia molecule from other ion.

Feature 8 is proposed by Wong et al [21] and defined as the ratio of number of peaks that have a relative intensity greater than 1% of total intensity to the total number of peaks in a spectrum. The reasoning for this feature is similar to that for Feature 1;

Feature 9 is proposed by Flikka et al [20] and defined as the standard deviation of delta mass (all mass differences between any two neighbor peaks) values in a spectrum. The reasoning for this feature is similar to that for Feature 4.

Feature 10 is proposed by Wu et al [6] and defined as the number of pairs of peaks whose m/z value difference is equal to the mass of a CO group or an NH group (all peaks are considered as single charged). This feature measures how likely one ion in a peptide CID mass spectrum is a supportive ion. Two kinds of supportive ions (a-ions and z-ions) were considered.

From the definitions and physical meaning of these features, the larger the values, the more likely the spectra are of high quality. Therefore, according to the feature values, each of these features can be used to assess the quality of tandem mass spectra and easily divided into two categories: one with high quality and another with poor quality. However, such individual assessments are not as good as the assessment from the combination of all 10 features [14,17].

### B. Integration of assessments based on individual features

**Figure 1:**
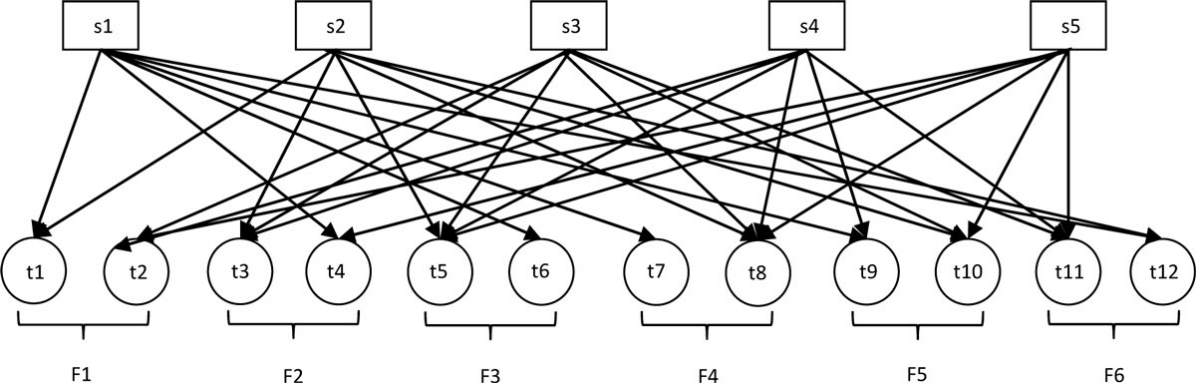
**An example of bipartite graph**.

In this section, we describe a method to integrate assessments based on each individual features into a consensus assessment. Based on each feature a dataset with *n *tandem mass spectra can be classified into two groups: one with high quality and one with poor quality. Therefore based on m features the dataset can be classified into *v *(=2m) groups in total. Each spectrum in the dataset must belong to m groups induced by m features. This formulates a natural bipartite graph representation as in Figure 1.

We use the following example to illustrate the bipartite graph representation and the problem formulation. Suppose we have a dataset consists of spectra {s1, s2, s3, s4, s5} and we use each of 6 features to classify them into 2 classes, respectively. The results are shown as in Table 1. Based on our model, the bipartite graph representation of these 6 features is shown in Figure 1. The spectral nodes are on the top side and group nodes are on the bottom side. In this bipartite graph, t1 represents the class 1, t2 the class 2 based on feature 1, and so on. Spectrum s3, for instance, belongs to group t2, t3, t5, t8, t10, t11 as it is in class 2 by Features 1, 4 and 5; and in class 1 by Features 2, 3 and 6.

**Table 1:** An object pool classified into several groups

Method/spectra	s1	s2	s3	s4	s5
F1	1	1	2	2	2
F2	1	1	2	2	2
F3	2	1	2	1	2
F4	1	2	1	2	2
F5	1	2	2	1	2
F6	2	1	1	2	2

In the proposed method, we will estimate the probabilities of *s_i _*(i = 1,...,n) being in the class of high quality or poor quality, these probabilities can be denoted by matrix *U_n×2_*. In our method, the probabilities of each group t_j _(j = 1,...,*v*) is also involved, which is denoted by matrix *Q_v×2_*. We have

$$u_{iz}=prob\left(s_{i}\text{ is in class z}\right)\;and\;q_{jz}=prob\left(t_{j}\text{ is class z}\right),$$

where z = 1 (means high quality) or 2 (means poor quality). Generally, a group *t_j _*corresponds to class *z *if the majority of spectra in this group belong to class *z*, meanwhile a spectrum belongs to class *z *if the majority of the groups it belongs to correspond to class *z*. Furthermore, the initial class labels for the groups can be denoted by matrix *Y_v×2 _*as *y_jz _*= 1 if the group *t_j_*' corresponds to class *z *and 0 otherwise. To estimate the probabilities in matrix *U*, we are about to optimize the following cost function with constraints [18]:

(2) $$\begin{array}{c} \text{min}J\left(U,Q\right)=\text{min}\left[\overset{k}{\underset{z=1}{\sum }}\overset{n}{\underset{i=1}{\sum }}\overset{v}{\underset{j=1}{\sum }}a_{ij}{\left(u_{iz}-q_{jz}\right)}^{2}+\alpha \overset{k}{\underset{z=1}{\sum }}\overset{n}{\underset{j=1}{\sum }}{\left(q_{jz}-y_{jz}\right)}^{2}\right]\\ s.t.\;\overset{k}{\underset{z=1}{\sum }}u_{iz}=1,\;\overset{k}{\underset{z=1}{\sum }}q_{jz}=1\\ u_{iz}\in \left[0,1\right],\;q_{iz}\in \left[0,1\right] \end{array}$$

where *a_ij _*is the (*i*, *j*) element of affinity matrix *A_n×v _*of the bipartite graph. It is defined as *a_ij _*= 1 if spectrum *s_i _*is assigned to the group *t_j _*and 0 otherwise. α is the positive parameter that expresses the confidence of the initial labels of the group nodes. This helps to avoid over-fitting. k = 2 is the number of consensus groups (with high quality or poor quality). As each spectra belongs to one of *k *groups by each of *m *features, we have

(3) $$\overset{v}{\underset{j=1}{\sum }}a_{ij}=m$$

It is obvious that the value of cost function is zero if all assessments based m individual features are perfect agreed. Nevertheless, this does not happen in practice. Therefore, the desired resultant matrix $Q_{\text{v}\times \text{k}}^{'}\;Q_{v\times k}^{'}$ will be obtained when the cost function in the constraint optimization proplem (2) reaches its minimal value. Finally, every spectrum will be assigned with a probability to class *z *directly according to the values in matrix $U_{\text{n}\times \text{k}}^{'}$.

From constraint optimization problem (2), we can see that for the given matrix U the objective function is quadratic in elements of matrix Q and that for the given matrix Q the objective function is quadratic in elements of matrix U. We therefore propose the following iterative algorithm to solve this optimization problem.

Step 1: Initialize Q by Y, that is, Q^t^=Y, and *t=*0.

Step 2: *t*=*t*+1,

Estimate *U^t ^*by solving

$$\underset{U}{\text{min}}J\left(U,Q^{t-1}\right)=\underset{U}{\text{min}}\left[\overset{k}{\underset{z=1}{\sum }}\overset{n}{\underset{i-1}{\sum }}\overset{v}{\underset{j=1}{\sum }}a_{ij}{\left(u_{iz}-q_{jz}^{t-1}\right)}^{2}+\alpha \overset{k}{\underset{z=1}{\sum }}\overset{n}{\underset{j=1}{\sum }}{\left(q_{jz}^{t-1}-y_{jz}\right)}^{2}\right]$$

to obtain

(4) $$u_{iz}^{t}=\frac{\overset{v}{\underset{j=1}{\sum }}a_{ij}q_{jz}^{t-1}}{\overset{v}{\underset{j=1}{\sum }}a_{ij}}=\frac{1}{m}\overset{v}{\underset{j=1}{\sum }}a_{ij}q_{jz}^{t-1}$$

Estimate *Q^t ^*by solving

$$\underset{Q}{\text{min}}J\left(U^{t},Q\right)=\underset{Q}{\text{min}}\left[\overset{k}{\underset{z=1}{\sum }}\overset{n}{\underset{i-1}{\sum }}\overset{v}{\underset{j=1}{\sum }}a_{ij}{\left(u_{iz}^{t}-q_{jz}\right)}^{2}+\alpha \overset{k}{\underset{z=1}{\sum }}\overset{n}{\underset{j=1}{\sum }}{\left(q_{jz}-y_{jz}\right)}^{2}\right]$$

to obtain

(5) $$q_{jz}^{t}=\frac{\overset{n}{\underset{i=1}{\sum }}a_{ij}u_{iz}^{t}+\alpha y_{jz}}{\alpha +\overset{n}{\underset{i=1}{\sum }}a_{ij}}$$

Step 3: Stop if ||*U^t ^*- *U^t^*^-1|| ^≤ *ε *and output *U*, where *ε *is a user specified small positive number.

In the above algorithm, we did not take the constraints in optimization problem (2). However, if the initial class labels for the groups Y_v×k _satisfy that

(6) $$\overset{k}{\underset{z=1}{\sum }}y_{jz}=1,\;y_{jz}\in \left[0,1\right]$$

the solutions of the above algorithm at every iteration t satisfying all constraints in optimization problem (2). We can use the technique of mathematical induction to prove that

**Theorem 1**: In the our algorithm if Eq(6) is true, the following is true

(7a) $$\overset{k}{\underset{z=1}{\sum }}u_{iz}^{t}=1,\;0\leq u_{iz}^{t}\leq 1,\;\;\text{for i}=1,\;\text{2},\ldots ,\;\text{n}$$

(7b) $$\sum_{z=1}^{k}q_{jz}^{t}=1,\;0\leq q_{jz}^{t}\leq 1,\;\;\text{for j}=1,2,\ldots ,\text{n}$$

for t = 1, 2, ......

Proof: for t = 1,

$$u_{iz}^{1}=\frac{\overset{v}{\underset{j=1}{\sum }}a_{ij}q_{jz}^{1-1}}{\overset{v}{\underset{j=1}{\sum }}a_{ij}}=\frac{1}{m}\overset{v}{\underset{j=1}{\sum }}a_{ij}q_{jz}^{0}=\frac{1}{m}\overset{v}{\underset{j=1}{\sum }}a_{ij}y_{jz}$$

It is obvious that $u_{iz}^{1}\geq 0$ and $u_{iz}^{1}=\frac{1}{m}\overset{v}{\underset{j=1}{\sum }}a_{ij}y_{jz}\leq \;\;\frac{1}{m}\overset{v}{\underset{j=1}{\sum }}a_{ij}=1$. Furthermore

$$\overset{k}{\underset{z=1}{\sum }}u_{iz}^{1}=\overset{k}{\underset{z=1}{\sum }}\frac{1}{m}\overset{v}{\underset{j=1}{\sum }}a_{ij}y_{jz}=\frac{1}{m}\overset{v}{\underset{j=1}{\sum }}a_{ij}\overset{k}{\underset{z=1}{\sum }}y_{jz}=\frac{1}{m}\overset{v}{\underset{j=1}{\sum }}a_{ij}=1$$

On the other hand

$$q_{jz}^{1}=\frac{\overset{n}{\underset{i=1}{\sum }}a_{ij}u_{iz}^{1}+\alpha y_{jz}}{\alpha +\overset{n}{\underset{i=1}{\sum }}a_{ij}}$$

as all values in this express are nonnegative and α is positive, it is true that $q_{jz}^{1}\geq 0$ and

$$q_{jz}^{1}=\frac{\overset{n}{\underset{i=1}{\sum }}a_{ij}u_{iz}^{1}+\alpha y_{jz}}{\alpha +\overset{n}{\underset{i=1}{\sum }}a_{ij}}\leq \frac{\overset{n}{\underset{i=1}{\sum }}a_{ij}+\alpha }{\alpha +\overset{n}{\underset{i=1}{\sum }}a_{ij}}=1$$

Furthermore

$$\overset{k}{\underset{z=1}{\sum }}q_{jz}^{1}=\overset{k}{\underset{z=1}{\sum }}\frac{\overset{n}{\underset{i=1}{\sum }}a_{ij}u_{iz}^{1}+\alpha y_{jz}}{\alpha +\overset{n}{\underset{i=1}{\sum }}a_{ij}}=\frac{\overset{n}{\underset{i=1}{\sum }}a_{ij}\overset{k}{\underset{z=1}{\sum }}u_{iz}^{1}+\alpha \overset{k}{\underset{z=1}{\sum }}y_{jz}}{\alpha +\overset{n}{\underset{i=1}{\sum }}a_{ij}}=\frac{\overset{n}{\underset{i=1}{\sum }}a_{ij}+\alpha }{\alpha +\overset{n}{\underset{i=1}{\sum }}a_{ij}}=1$$

Assume that for t=r, Eqs (7a) and (7b) are true, that is

(8a) $$\sum_{z=1}^{k}u_{iz}^{r}=1,0\leq u_{iz}^{r}\leq 1,\;\text{for i}=\text{1},\text{2},\ldots ,\text{n}$$

(8b) $$\sum_{z=1}^{k}q_{jz}^{r}=1,0\leq q_{jz}^{r}\leq 1,\;\text{for j}=\text{1},\text{2},\ldots ,\text{n}$$

Then t=r+1, from our algorithm it follows

(9a) $$u_{iz}^{r+1}=\frac{\overset{v}{\underset{j=1}{\sum }}a_{ij}q_{jz}^{r}}{\overset{v}{\underset{j=1}{\sum }}a_{ij}}=\frac{1}{m}\overset{v}{\underset{j=1}{\sum }}a_{ij}q_{jz}^{r},$$

(9b) $$q_{jz}^{r+1}=\frac{\overset{n}{\underset{i=1}{\sum }}a_{ij}u_{iz}^{r+1}+\alpha y_{jz}}{\alpha +\overset{n}{\underset{i=1}{\sum }}a_{ij}}$$

From Eq (8b) and (9a) it follows

(10a) $$\sum_{z=1}^{k}u_{iz}^{r+1}=1,\;0\leq u_{iz}^{r+1}\leq 1,\;\text{for i}=\text{1},\text{2},\ldots ,\text{n}$$

Furthermore, from (9b) and (10a) it follows

(10b) $$\sum_{z=1}^{k}q_{jz}^{r+1}=1,\;0\leq q_{jz}^{r+1}\leq 1,\;\text{for j}=\text{1},\text{2},\ldots ,\text{n}$$

Therefore, for any positive integer t, (7a) and (7b) are true.

**Theorem 2**: From our algorithm it follows that

(11) $$J(U^{t},Q^{t})\geq J(U^{t+1},Q^{t+1})\;\text{for t}=\text{1},\text{2},\ldots \ldots .$$

*Proof*: from the algorithm, it follows for t = 1,2,....

$$J(U^{t},Q^{t})\geq \underset{U}{\min}J(U,Q^{t})=J(U^{t+1},Q^{t})\geq \underset{Q}{\min}J(U^{t+1},Q)=J(U^{t+1},Q^{t+1})$$

From inequality above, *J *(*U^t ^*, *Q^t ^*) is non-increase as the number of iteration t is increasing. On the other hand, *J *(*U^t ^*, *Q^t ^*) is bounded below. Therefore, $\underset{t\to \infty }{\text{lim}}J\left(U^{t},Q^{t}\right)$ exists, that is, our algorithm is converged.

The algorithm reflects that at each iteration the probability estimation of group node Q receives the information from its neighboring spectral nodes while not deviating from its initial value Y too wild. In return, the updated probability estimates of group nodes propagate the information back to its neighboring spectral nodes. The propagation stops when the process converges. The process converges to a stationary point.

### Experimental results and discussions

To evaluate our proposed method, experiments are conducted on two low resolution tandem mass spectral datasets: TOV and ISB.

#### TOV dataset

The tandem mass spectra in this dataset are acquired from a LCQ DECA XP ion trap spectrometer (ThermoElectron Corp.) as described in [19]. The number of spectra in this dataset is 22, 576, and these spectra are searched using SEQUEST against the ipi.HUMAN. v3.42.fasta containing 72, 340 protein sequences and 5 contaminant sequences.

#### ISB dataset

The spectra in this dataset are acquired from the complex of 18 control mixture proteins which were analyzed by mLC-MS on an ESI-ITMS (ThermoFinnigan, San Jose, CA) using a standard top-down data-dependent ion selection approach [4]. This dataset consists of 37, 044 tandem mass spectra. These spectra were searched against a human protein database appended with the sequences of the 18 standard proteins and other common contaminants (totally, 5, 395 protein sequences in the final database) using SEQUEST search program.

The distribution of tandem spectra is shown in Table 2. 'H' represents the number of the high quality spectra, and 'P' represents the number of the poor quality spectra. The assignments of spectra were determined by SEQUEST score with the cut-off score of 2.8. Spectra with score less than threshold were labeled as poor quality spectra; otherwise, they were labeled as high quality spectra.

**Table 2:** The distribution of multiply charged spectra in the ISB and TOV dataset

	H	P	Total
TOV	1136	21440	22576
ISB	1047	35997	37044

In the experiment, we applied the proposed method on both datasets to obtain assessments based on individual features. For each feature, spectra with the top 50% feature values are assigned to high quality class. In the method, the parameter α in the model was taken as 90.

**Figure 2:**
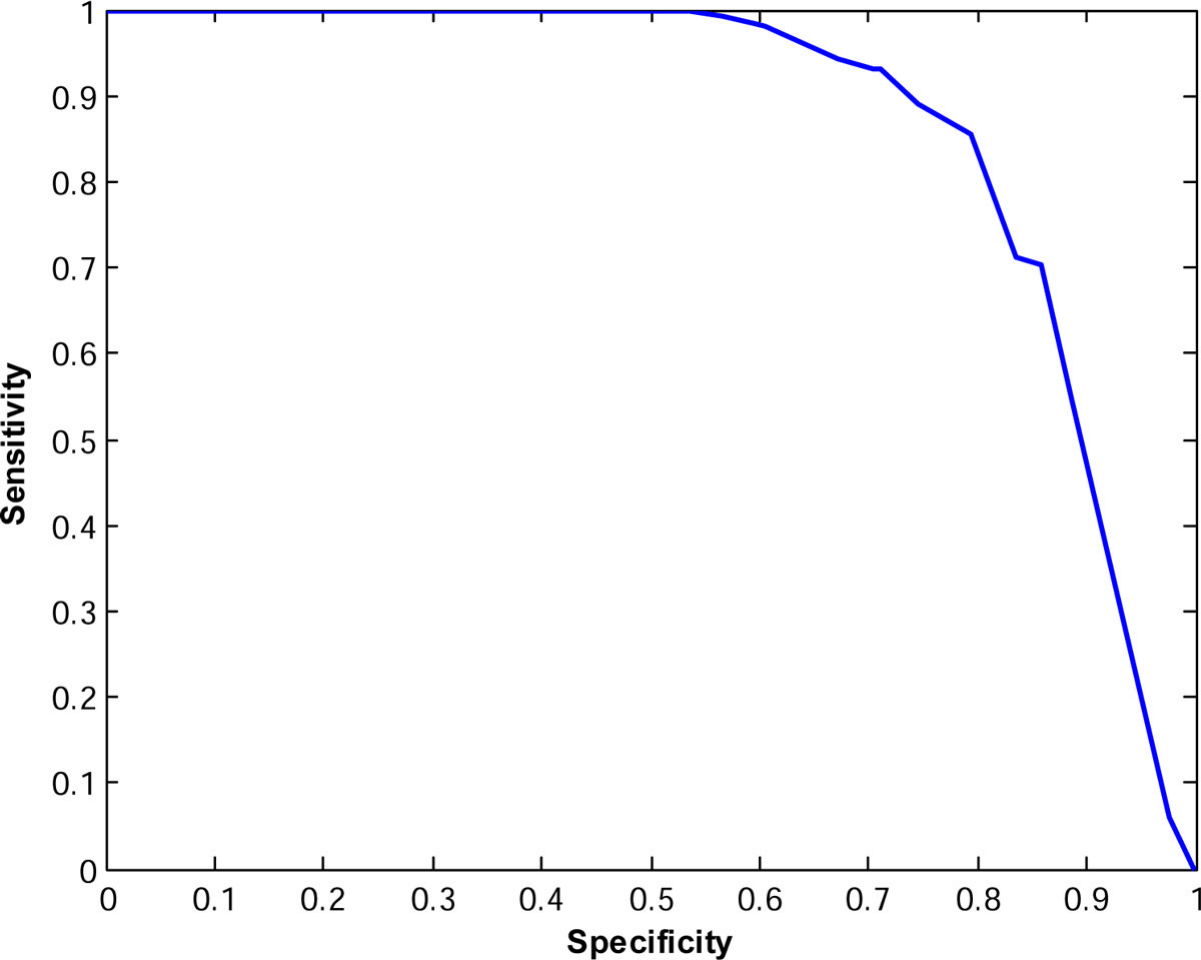
**ROC curve for the proposed classifier for TOV spectra**.

**Figure 3:**
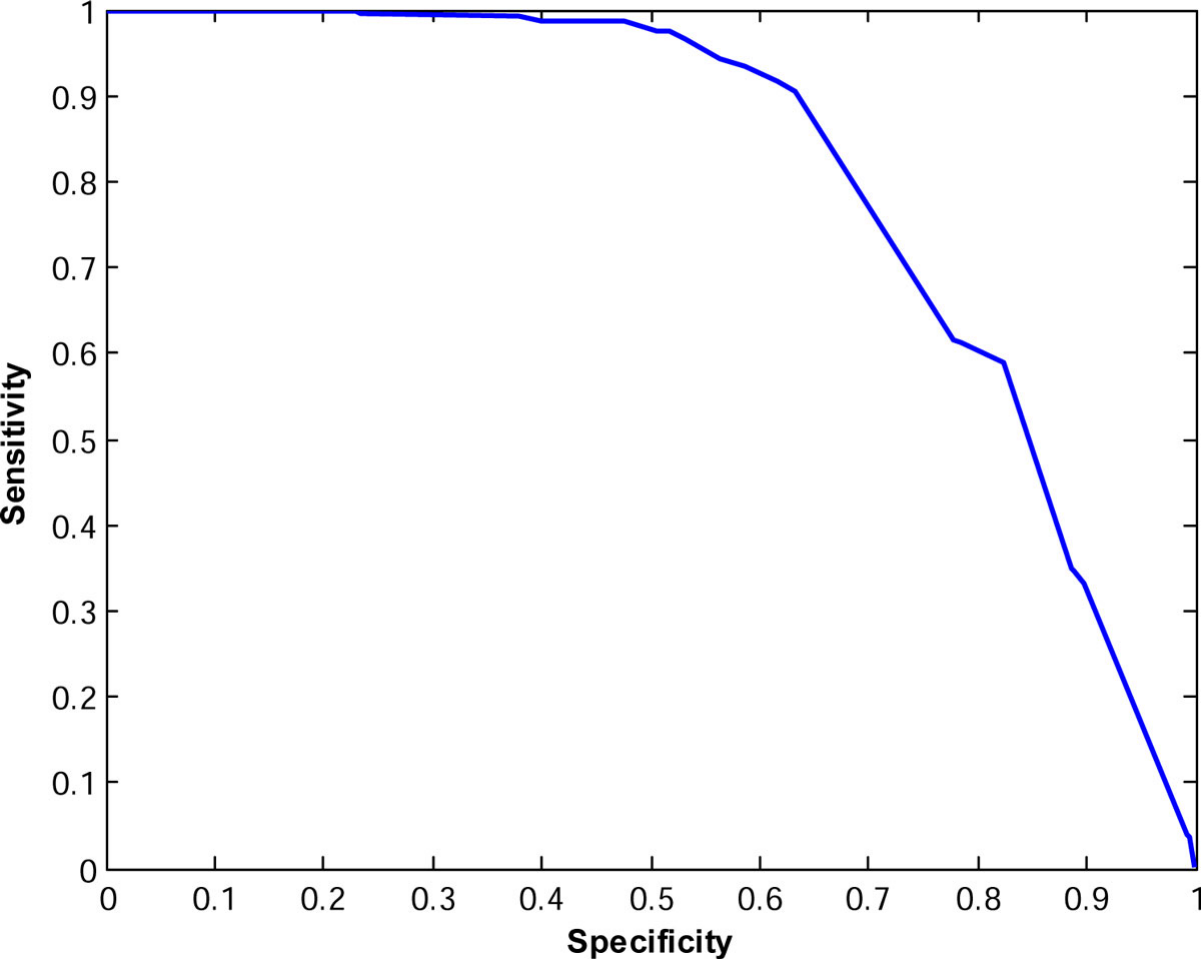
**ROC curve for the proposed classifier for ISB spectra**.

Figures 2 and 3 show the ROC curves for the consensus classifiers for TOV and ISB datasets, respectively. For TOV dataset, the proposed method can eliminate about 74% of the poor quality spectra while only losing less than 9% of the high quality spectra at the best case. For the ISB dataset, the proposed method can filter out about 63% of poor quality spectra while only losing 10% of high quality spectra. If we just search the TOV and ISB spectra in the high-quality group with SEQUEST, we can save about 56% (= 1-10042/22576) and 62% (= 1-14087/37044) of searching time while losing only about 10% of the interpretable spectra. These results indicate that our proposed method in this paper outperforms the method in [16].

Furthermore, our method achieved a better result from TOV dataset than the one from ISB dataset. This may because that there are more poor quality spectra in ISB dataset (35997/37044 = 97%) than in TOV dataset (21440/22576 = 95%). High percentage of poor quality spectra makes quality assessment more challenging [17]. Another reason maybe is that there are more triply charged spectra in ISB dataset (18044) than in TOV (9732). Triple charged spectra contain more doubly charged peaks than both doubly and singly charged spectra. The quality of triply charged spectra are not well described by 10 features we used in this paper, especially, feature 3, 6, 7, 10 we used are only designed for singly charged peaks while triply charged spectra produce many doubly charged peaks [25,26].

## Conclusions and future work

This paper has presented an un-supervised machine learning method to integrate the assessments based on individual features (which is easy to do with a low precision) into a consensus assessment with a higher precision. This unsupervised machine learning method first estimate the conditional probability of a spectrum being high quality from the assessments based on individual features. The estimation of the probabilities is solved through a constraint optimization problem. Experiment results illustrate that if we just search spectra assessed as the high-quality in TOV and ISB, we can save about 56% and 62% of searching time while losing only 9% and 10% of high-quality spectra, respectively. This result indicates that the proposed method is useful in saving database searching time. Besides, under the true positive rate (90%), our new method reaches the true negative rate at 74% and 63%, respectively. This indicates that the new method has a good performance on quality assessment of tandem mass spectra. Also, this result shows the way we estimate the conditional probability is effective.

However, the proposed method could be improved in several ways for the future work. For example, in the ten features we adapted, four of them were calculated for singly charged peaks. This makes the classification method less effective on the triply or higher charged spectra. In the future, we may adapt different features for different charges of spectra. In this study, the value of *α *and percentage cut-off value for individual features were taken according to several trial and error repeats. In the future, a more objective method should be developed for specifying these values. In addition, the proposed constraint optimization model can be applied for other unsupervised classification problems in bioinformatics and proteomics.

## Competing interests

The authors declare that they have no competing interests.

## Authors' contributions

WL drafted the paper, ran the program to compute the feature values, and ran and wrote the program of the iterative algorithm for the constraint optimization problem. JW and FXW developed the iterative algorithm for the constraint optimization problem, proved its convergence, and substantially modified the paper draft. FXW and WJZ initiated and supervised this research work. All authors read and approved the final manuscript.
